# The Cyclophilin-Binding Agent Sanglifehrin A Is a Dendritic Cell Chemokine and Migration Inhibitor

**DOI:** 10.1371/journal.pone.0018406

**Published:** 2011-03-31

**Authors:** Sabrina N. Immecke, Nelli Baal, Jochen Wilhelm, Juliane Bechtel, Angela Knoche, Gregor Bein, Holger Hackstein

**Affiliations:** 1 Institute for Clinical Immunology and Transfusion Medicine, Justus-Liebig-University Giessen, Giessen, Germany; 2 Department of Pathology, Justus-Liebig-University Giessen, Giessen, Germany; Statens Serum Institute, Denmark

## Abstract

Sanglifehrin A (SFA) is a cyclophilin-binding immunosuppressant but the immunobiology of action is poorly understood. We and others have reported that SFA inhibits IL-12 production and antigen uptake in dendritic cells (DC) and exhibits lower activity against lymphocytes. Here we show that SFA suppresses DC chemokine production and migration. Gene expression analysis and subsequent protein level confirmation revealed that SFA suppressed CCL5, CCL17, CCL19, CXCL9 and CXCL10 expression in human monocyte-derived DC (moDC). A systems biology analysis, Onto Express, confirmed that SFA interferes with chemokine-chemokine receptor gene expression with the highest impact. Direct comparison with the related agent cyclosporine A (CsA) and dexamethasone indicated that SFA uniquely suppresses moDC chemokine expression. Competitive experiments with a 100-fold molar excess of CsA and with N-Methyl-Val-4-cyclosporin, representing a nonimmunosuppressive derivative of CsA indicated chemokine suppression through a cyclophilin-A independent pathway. Functional assays confirmed reduced migration of CD4+ Tcells and moDCs to supernatant of SFA-exposed moDCs. Vice versa, SFA-exposed moDC exhibited reduced migration against CCL19. Moreover, SFA suppressed expression of the ectoenzyme CD38 that was reported to regulate DC migration and cytokine production. These results identify SFA as a DC chemokine and migration inhibitor and provide novel insight into the immunobiology of SFA.

## Introduction

The immunophilin-binding agents cyclosporine A (CsA), FK506 and rapamycin represent potent immunosuppressive agents that have revolutionized bone marrow and solid organ transplantation as well as treatment of autoimmune diseases. Sanglifehrin A (SFA) is a novel immunophilin-binding immunosuppressive drug isolated from the actinomycetes strain Streptomyces A92-308110 exhibiting high affinity binding to Cyclophilin A, but unknown mechanism of action [1]–[4]. SFA does not affect the calcineurin phosphatase or the mammalian target of rapamycin and it does not inhibit purine or pyrimidine de novo synthesis [5]. Crystal structure analysis of SFA in complex with cyclophilin A indicated that the effector domain of SFA exhibits a chemical and three-dimensional structure very different from CsA suggesting different immunosuppressive action [6].

In contrast to CsA, the immunobiology of SFA is not well understood. Previous reports demonstrated that SFA is different from known immunosuppressive agent [5], [7]. SFA is approximately 15–35-fold less potent than CsA at inhibiting T cell proliferation in mouse and human MLR cultures [5]. In contrast to CsA and FK506, SFA does not inhibit TCR-induced anergy [8]. Similarly to rapamycin, SFA blocks IL-2 dependent proliferation in T cells [5].

Different groups have reported that SFA exerts suppressive effects on human and mouse DC. SFA suppresses antigen uptake, IL-12 and IL-18 production of DC in vitro and in vivo but it does not inhibit DC differentiation and surface costimulatory molecule expression [9]–[12].

DCs are professional antigen presenting cells that play a central role in the initiation and modulation of innate and adaptive immunity [13]–[17]. DC attract effector cells through different chemokines that are critical for the coordination of the sequential interaction of immediate effector cells, such as neutrophils and natural killer cells and the delayed activation of antigen-specific B and T lymphocytes [18]–[19]. Immunophilin-binding immunosuppressive agents, especially rapamycin, and to a lesser extent, CsA, have been reported to target key functions of DC [13], [20]. Rapamycin has been demonstrated to inhibit functional maturation of DC and to promote their tolerogenicity in different animal models [21]–[24].

In an experimental transplant model, SFA monotherapy did not suppress acute organ allograft rejection supporting the hypothesis that it does not represent a primary T cell inhibitor [11]. Interestingly, in combination with CsA, SFA efficiently promoted long-term allograft survival [11]. Furthermore, in a chronic allograft rejection model [11], addition of SFA to CsA-treated recipients markedly inhibited chronic rejection compared to animals receiving high dose CsA monotherapy, suggesting that SFA exerted unique immunobiological effects different from inhibition of calcineurin phosphatases.

Current knowledge indicates that SFA represents a novel class of immunophilin-binding metabolite both with respect to chemical structure and functional activity [5]–[6], [9]–[11]. There is a paucity of knowledge about the immunobiological effects of SFA since each study focused on selected functions or selected aspects with professional antigen presenting cells being either directly or indirectly involved. Systematic studies investigating the effects of SFA are completely lacking.

In this report we describe the results of the first systematic analysis of the immunobiological effects of the novel immunophilin-binding agent SFA on human monocyte-derived DC (moDC) using a combination of genome-wide expression profiling with subsequent confirmation on the protein level and functional in vitro and in vivo assays. Results indicate that SFA represents a novel DC chemokine and migration inhibitor.

## Results

### Sanglifehrin A blocks chemokine gene expression in human moDCs

To systematically identify specific gene expression changes by SFA, we compared human moDCs cultured in the presence of either vehicle or drug. Human DCs were generated by differentiation of monocytes with GM-CSF and IL-4 for five days. On day 5 human moDCs were either treated with 1 µM SFA or vehicle for 1 hour followed by 12 h LPS stimulation. The RNA of SFA vs. vehicle-treated human moDCs was analysed by whole genome Oligo Microarray. The Microarray data showed 260 significantly regulated genes . 190 genes are significantly down regulated and 70 genes are up-regulated. The complete data are deposited in the Gene Expression Omnibus database (http://www.ncbi.nlm.nih.gov/geo/) with the GSE15956 accession number.

### Pathway analysis of SFA vs. vehicle differentially expressed genes

**Figure 1:**
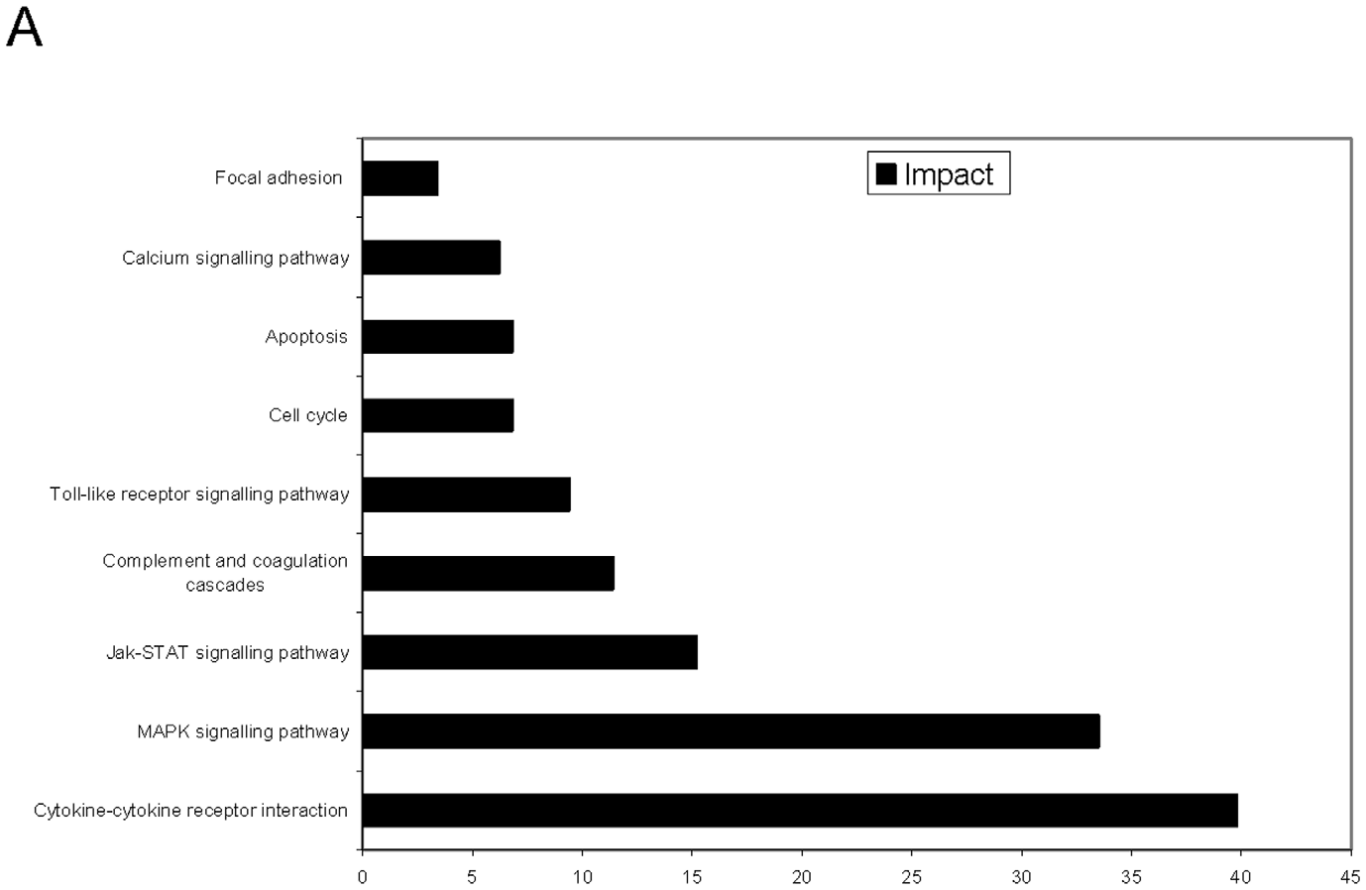
SFA inhibits cytokine-cytokine receptor interactions. Human moDCs were treated with 1 µM SFA or vehicle for 1 hour and total RNA was prepared after 12 h stimulation with 1 µg/mL LPS. The cDNA were labelled with Cy3- and Cy5-fluorescent dyes for microarray hybridization. Chart summarizes the results of the pathway impact analysis. Numbers indicate the impact factor. The impact factor is calculated based on the normalized fold of gene expression change, the number and amount of perturbation of genes downstream from it and the proportion of differentially regulated genes in the respective pathway [25].

We analyzed the gene expression changes with PathwayExpress from OntoExpress (http://vortex.cs.wayne.edu/ontoexpress/) to get information about the biological functions [25]. Cytokine-cytokine-receptor interaction, MAPKinase-, JAK/STAT-signalling pathway and complement and coagulation cascades are the functional groups containing the largest number of identified proteins (Figure 1). The highest impact factor with 39.8 was found with respect to cytokine-cytokine-receptor interactions. Analysis of the cytokine pathway subfamilies revealed that SFA interfered most frequently with the chemokine subfamily. Seven out of eleven significantly regulated cytokines were chemokines (Table 1).

**Table 1:** SFA-regulated genes in the cytokine-cytokine receptor pathway.

systematic name	human ligand	coefficient	p.value	Iods*
CCL1	I-309	2.47	3.66E-07	7.36
CCL17	TARC	−2.41	4.89E-09	9.76
CCL19	MIP-3β, ELC, exodus-3	−3.61	4.61E-07	7.14
CCL26	Eotaxin-3	1.58	9.72E-07	6.40
CCL5	RANTES	−2.92	1.78E-09	11.8
CXCL10	IP-10	−1.27	7.6183E-05	1.88
CXCL9	MIG	−2.96	6.92E-08	8.94
EBI3		−3.08	3.81E-08	9.47
IL1R2		2.84	3.78E-08	9.48
TNFRSF14		−2.11	3.28E-07	7.47
TNFRSF4		−1.54	1.07E-06	6.31

*lods (log odds ratio for differential expression given an expectation of 1% regulated genes on the array.

### SFA suppresses CCL5, CCL17, CCL19, CXCL9 and CXCL10 production by human moDCs at the protein level

**Figure 2:**
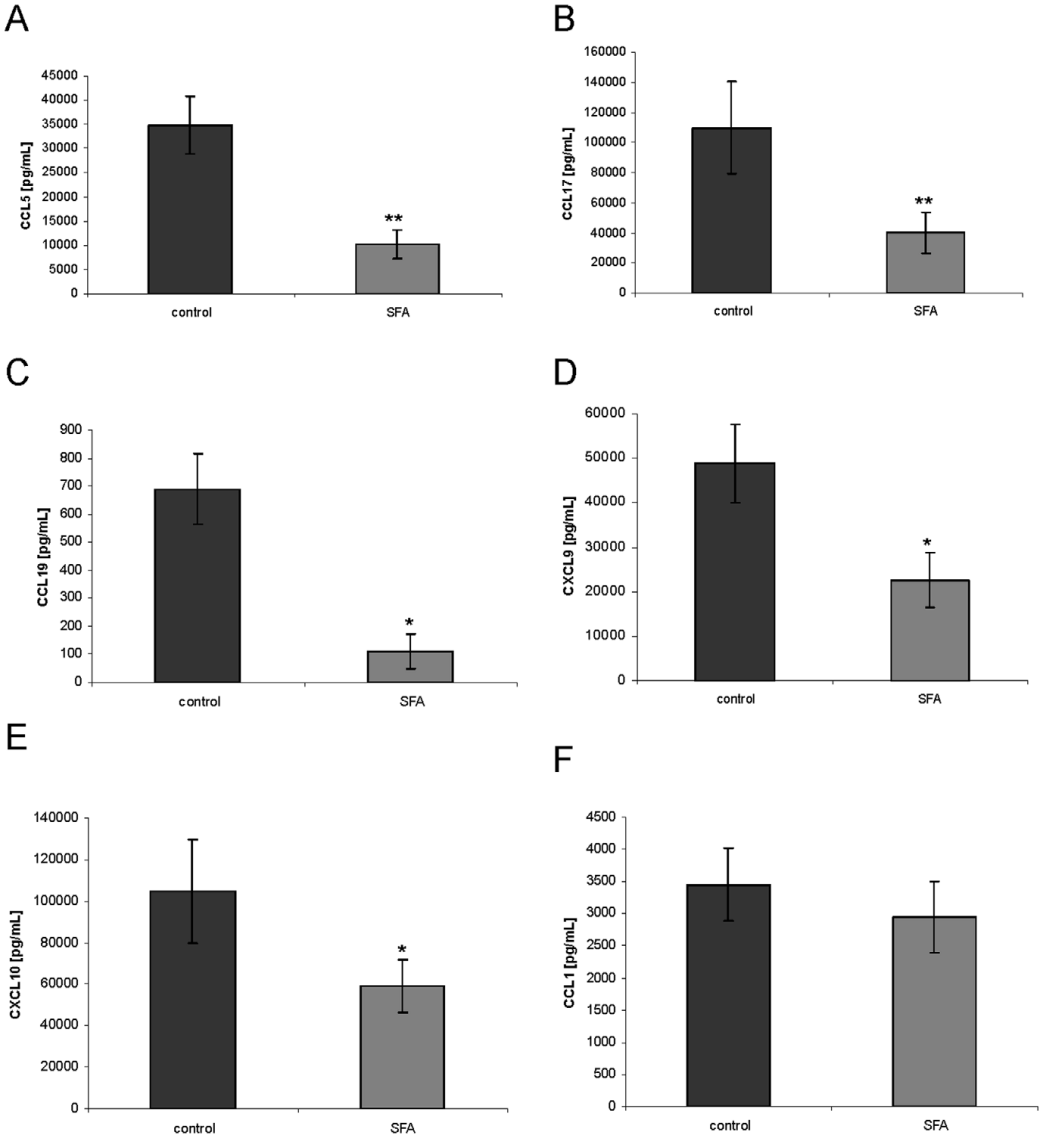
Rapid suppression of moDC chemokine production by SFA. Human moDCs were generated in the presence of GM-CSF and IL-4. 100 nM SFA or drug vehicle were added 4 h before stimulation with 100 ng/mL LPS. 12 hours later the supernatant was collected. CCL5 (A), CCL17 (B), CCL19 (C), CXCL9 (D), CXCL10 (E) and CCL1 (F) were analyzed by ELISA. The results are representative of n = 6 (A,D), n = 7 (E, F), n = 8 (C), and n = 11 (B) independent experiments (Mean ± SEM). **p<0.01, *p<0.05 versus vehicle.

To confirm chemokine suppression by SFA in moDCs, we analysed protein expression by ELISA. Short-term treatment (4 h) of human moDCs with 100 nM SFA resulted in significant suppression of CCL5, CCL17, CXCL9 and CXCL10 production (Figure 2 A, B, D, E). 100 nM SFA inhibited 84% of CCL19 production compared with vehicle-exposed controls (Figure 2C). The up-regulation of CCL1 gene expression could not be confirmed at the protein level (Figure 2 F). CCL26 expression was below the detection limit of the ELISA (data not shown).

### Rapid dose dependent moDC chemokine suppression through SFA

**Figure 3:**
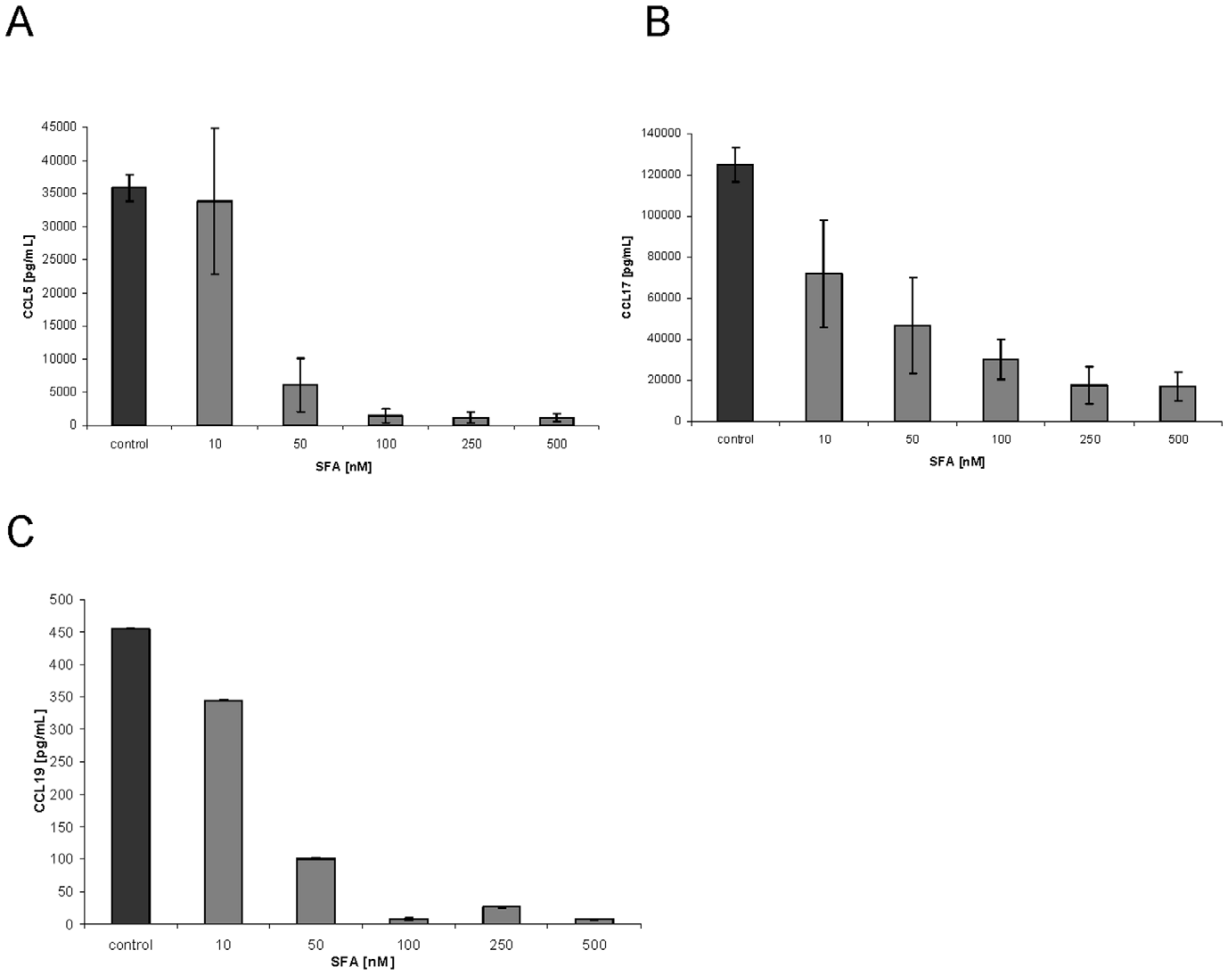
Dose-dependent suppression of CCL5, CCL17 and CCL19 in moDCs by SFA. Human moDC were exposed on day 5 with 10, 50 100, 250 and 500 nM of SFA and 4 hours later stimulated with 100 ng/mL LPS. CCL5 (A), CCL7 (B) and CCL19 (C) production were analyzed after 12 h stimulation by ELISA. Mean (± SEM) of n = 3 (A) and n = 4 (B, C) independent experiments.

Having established that short-term SFA treatment of moDC significantly inhibited chemokine gene and protein expression we next analysed the potency of SFA to suppress CCL5, CCL17 and CCL19 in moDC. Administration of 50 nM SFA for 4 h resulted in >80% CCL5 suppression (Figure 3 A), and 100 nm SFA resulted in >70–90% CCL17 and CCL19 suppression (Figure 3 B, C).

### Unique chemokine inhibition by SFA in comparison to CsA and dexamethasone

**Figure 4:**
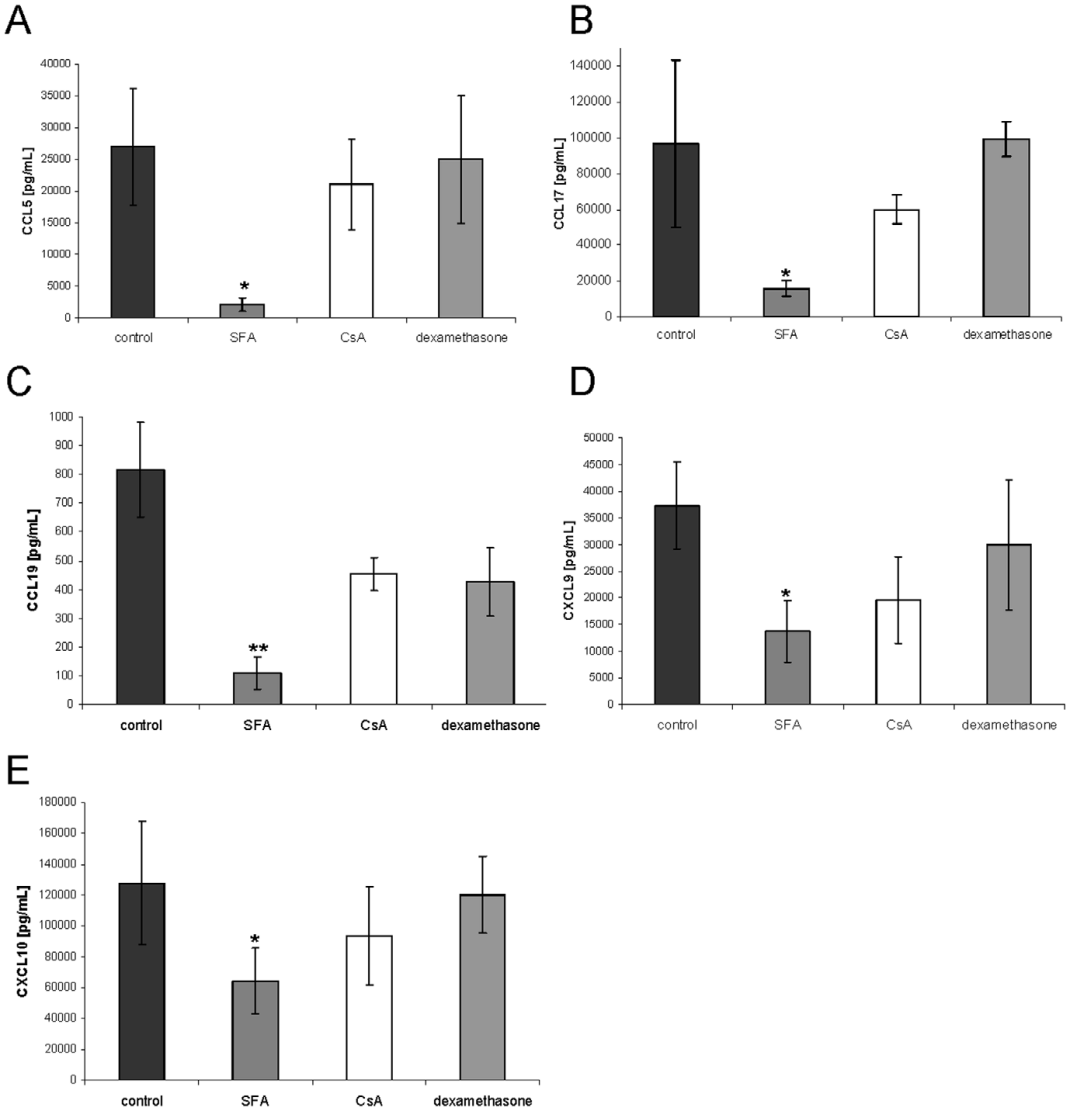
moDC chemokine suppression by SFA is unique in direct comparison to CsA and dexamethasone. Human moDC were exposed to 100 nM SFA, CsA, dexamethasone or vehicle. After 4 h moDCs were stimulated with LPS (100 ng/ml) and CCL5, CCL17, CCL19, CXCL9, CXCL10 were analyzed by ELISA after 12 h. In contrast to CsA and dexamethasone, SFA markedly inhibited CCL5 (A), CCL17 (B) and CCL19 expression (C).

Both, SFA and CsA bind with high affinity to cyclophilin in cells [5]. Dexamethasone is a synthetic member of the glucocorticoid class of steroid hormones and represents a prototypic immunosuppressive agent. 100 nM CsA and dexamethasone only moderately affected CCL5, CCL17, CCL19, CXCL9 and CXCL10 production by moDC (Figure 4 A–E). In contrast, 100 nM SFA inhibited CCL5, CCL17, and CCL19 production in moDCs (Fig. 4 A–C). In contrast to SFA, CsA did not exhibit dose dependent effects on CCL5, CCL17 and CCL19 expression up to suprapharmacological doses of 10 µM . These experiments suggested that SFA is a novel pleiotropic DC chemokine inhibitor exhibiting a unique inhibitory profile when compared to the related cyclophilin-bnding agent CsA and dexamethasone.

### SFA inhibits chemokine production in moDCs in a cyclophilin-A independent manner

**Figure 5:**
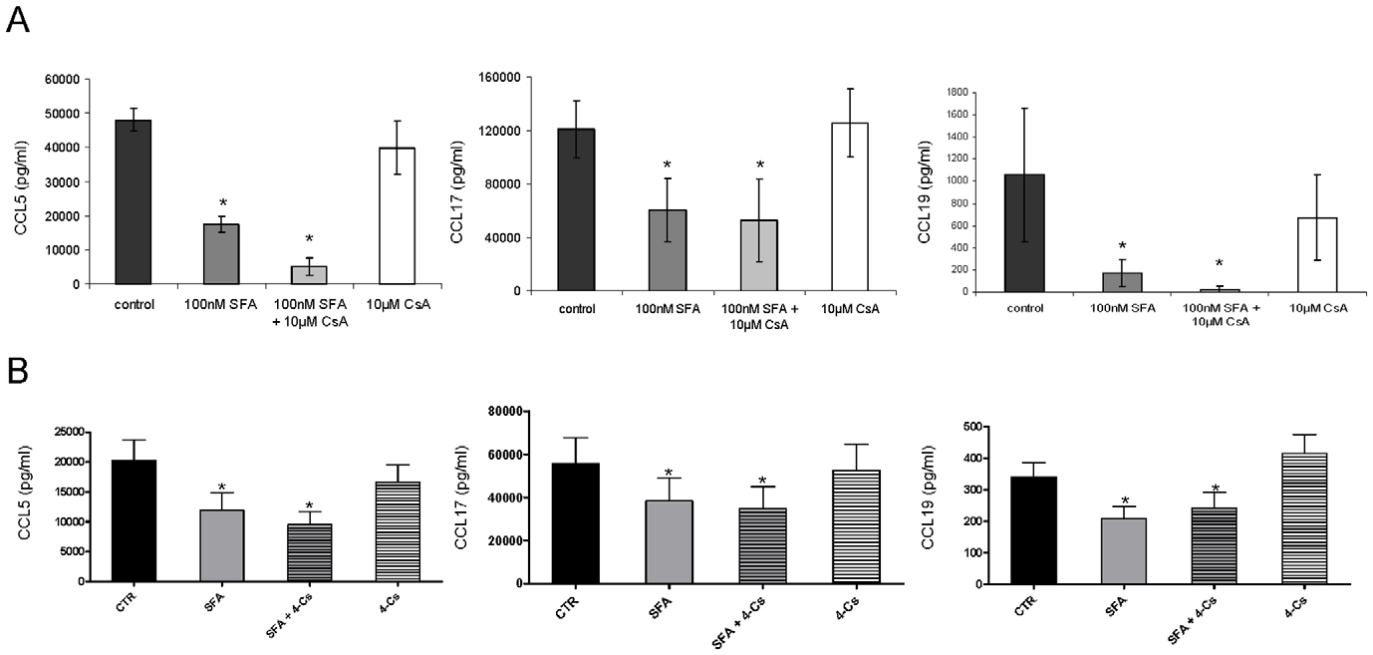
moDC chemokine suppression by SFA is independent of cylophilin A binding. To analyze whether DC chemokine suppression by SFA is dependent on cyclophilin A binding competitive experiments with a 100-fold molar excess of CsA (A) and with a 10-fold molar excess of a cyclophilin-binding nonimmunosuppressive derivative of CsA, 4_Cs (B) were performed. 4-Cs has been reported to efficiently inhibit SFA cyclophilin A binding [5]. Human moDC were pre-exposed to 10 µM CSA and 1 hour later to 100 nM SFA (A). With respect to 4-Cs, moDC were pre-exposed to 2500 nM 4-Cs and 1 hour later to 250 nM SFA (B). After 4 hours, moDCs were stimulated with LPS (100 ng/ml) and DC chemokine production were analyzed after 12 h by ELISA. In the presence of a 100-fold molar excess of CsA or a 10-fold molar excess of 4-Cs, SFA's DC chemokine inhibitory activity was not abrogated. In contrast, 10 µM CSA did not inhibit CCL5 (F) or CCL17 (G) moDC production and only moderately inhibited CCL19 (H) expression. 4-Cs did not inhibit CCL5, CCL17 or CCL19 production. Mean (± SEM) of n = 3–6 independent experiments. **p<0.01, *p<0.05 versus vehicle.

To address the question whether SFA's inhibitory activity on chemokine expression is dependent on cyclophilin A binding, we performed competitive experiments with a 100-fold molar excess of CsA (10 µM). CsA has been described to potently inhibit the binding of SFA to cyclophilin A [5] and we have found that CsA, in contrast to SFA, did not abrogate CCL5, CCL17 and CCL19 production in moDCs. moDCs were preincubated for 1 hour with 10 µM CsA in order to saturate cylophilin binding sites. Whereas even 10 µM CsA did not exert major effects on CCL19 production in human moDC, addition of 100 nM SFA one hour later markedly inhibited CCL19 expression (Figure 5A). Similar results were obtained with respect to CCL5 and CCL17 expression (Figure 5A). These results indicated that chemokine suppression by SFA is independent on cyclophilin A binding since binding of CsA to cyclophilin A did not abrogate or impair the activity of SFA. Interestingly, we observed that a combination of suprapharmacological doses of CsA with low doses of SFA consistently improved to some extent the suppressive activity of SFA (Figure 5A). These data might indicate that preincubation with CsA can possibly alter the binding stochiometry of SFA to other immunophilins/target molecules resulting in different immunosuppressive activity. However, since competitive experiments with CsA exhibited technical limitations, especially the fact that CsA itself exerts immunosuppressive activity, we performed additional experiments with a cyclophilin-binding non-immunosuppressive derivative of CsA, 4-Cs that potently inhibits the binding of SFA to cyclophilin A [5]. The results indicated that addition of 4-Cs to moDC cultures did not abrogate the suppressive activity of SFA (Figure 5B) suggesting that DC chemokine suppression by SFA was independent of cyclophilin binding.

### Inhibition of moDC and CD4^+^ T cell migration through supernatant from SFA-exposed moDC

**Figure 6:**
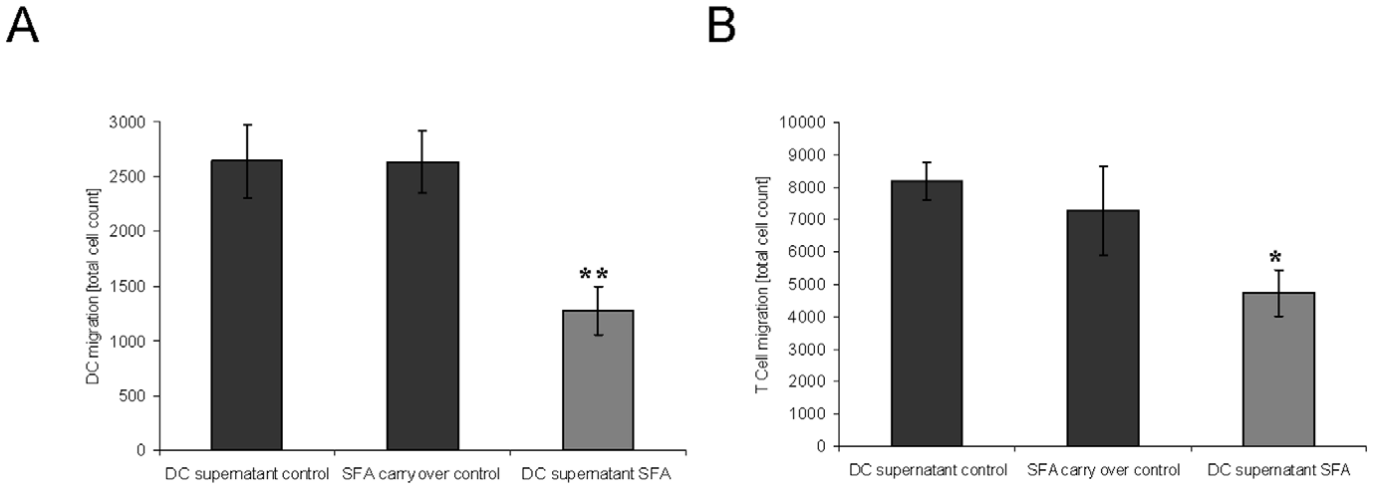
Supernatant of SFA-exposed moDC induces reduced migration of activated moDC and CD4+ T cells. moDCs were generated in the presence of GM-CSF and IL-4 and activated for 12 h with LPS. CD4 T cells were isolated by microbead-sorting and activated for 16 h with CD3/CD28 mAbs. DC supernatant from SFA- or vehicle-exposed moDCs was harvested 12 h after LPS activation and added to the lower chamber of the transwell. The “SFA carry over control” consisted of supernatant of control-treated moDCs+1 µM SFA. Migration of cells was quantitated by flow cytometry. (A) Activated moDCs were inserted in the upper chamber of the transwell and migration was analysed after 4 h. (B) Activated CD4^+^-T cells were set in the upper chamber of the transwell and migration was analysed after 4 h. The spontaneous migration of cells was subtracted from the results (mean DCs: 1673; T cells: 8676). The results are representative for n = 9 (A) and n = 5 (B) independent experiments. Mean (± SEM) **p<0.01, *p<0.05 versus vehicle.

To confirm the functional relevance of SFA's inhibition of moDC chemokine expression we analysed CD4^+^ T cell migration and moDC migration towards supernatant from SFA-exposed maturing moDCs and vehicle-exposed controls (Figure 6 A). To eliminate any possibility of a direct influence of SFA on migration, we added 1 µM SFA to the supernatant of vehicle-treated moDCs and included these “SFA carry over controls” in the experiments. These experiments revealed significant inhibition of both moDC migration and, independently, CD4^+^ T cell migration towards supernatant from maturating, SFA-exposed moDCs (Figure 6).

### SFA inhibits moDC migration towards CCL19 in a CCR7 independent manner

**Figure 7:**
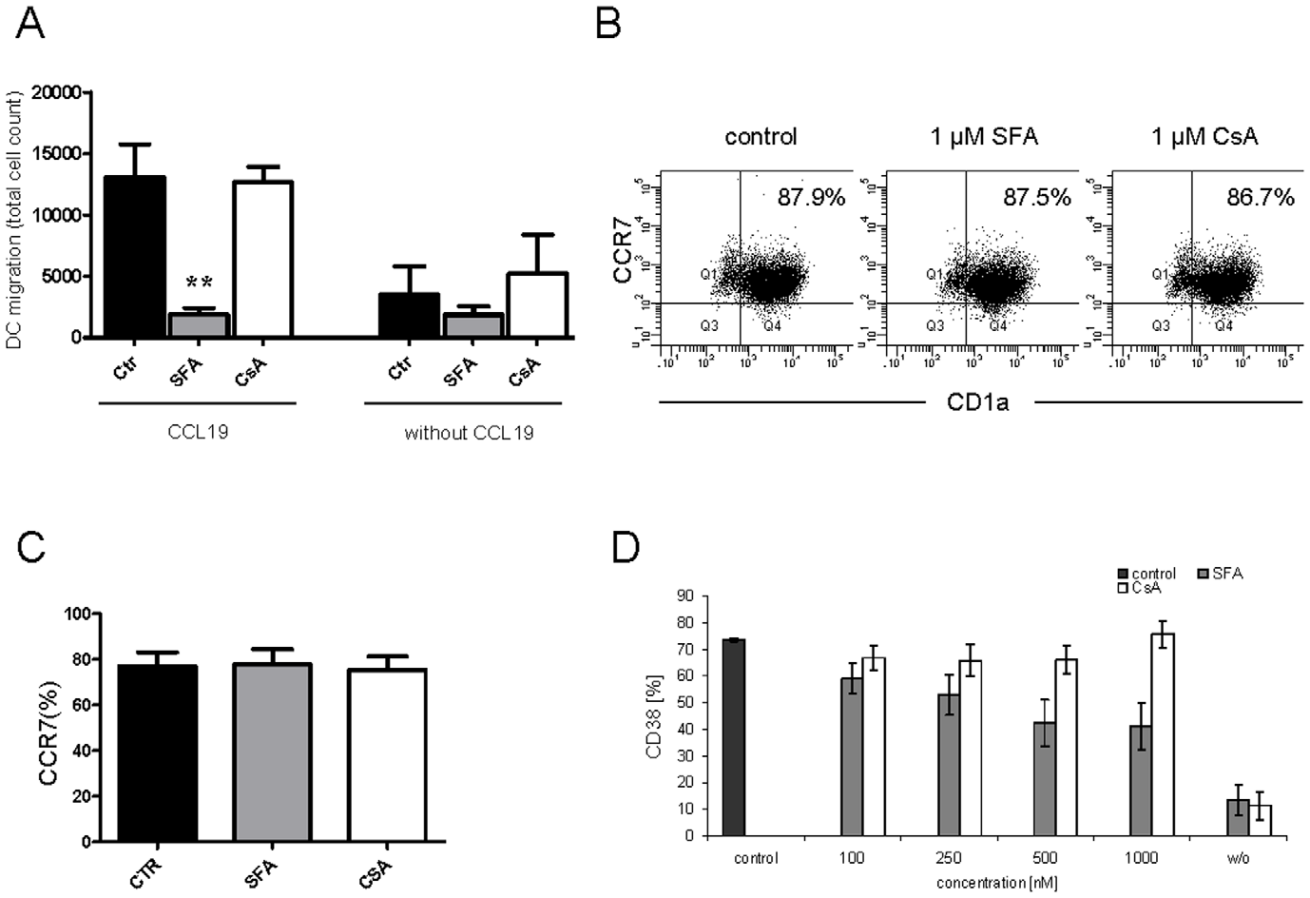
SFA suppresses moDC migration to CCL19 in a CCR7 independent manner and inhibits CD38 expression. Human moDCs were exposed to SFA, CsA or vehicle and matured for 12 h (A) or 24 h (B–D) with LPS (100 ng/ml). (A) SFA-exposed moDC (1 µM), CsA-exposed moDC (1 µM) or control moDC were added to the upper chamber of the transwell and migrated to CCL19 in the lower chamber as described in Materials and Methods. Control experiments included the spontaneous migration in the absence of CCL19. The results indicate number of migrated DC (mean ± SEM). (B–DF) Surface CCR7 and CD38 expression of human moDCs exposed to 1 µM SFA, 1 µM CsA (B, C) or drug vehicle was analyzed by flow cytometry with mAbs. (D) SFA but not CsA inhibits CD38 expression on matured CD1a+ moDC. The results are representative for of n = 6 (A) and n = 3 (B–C) independent experiments (mean ± SEM). The results in D are representative for n = 5–8 (100–500 nM SFA, CsA) and n = 2 (1000 nM SFA, CsA) independent experiments *p<0.05 ; **p<0.01 versus drug-vehicle.

Given the fact that SFA efficiently inhibited chemokine production by human moDCs we next questioned whether SFA also directly inhibits moDC migration of maturing DCs (Figure 7 A). The capacity of SFA-treated LPS-matured human moDCs to migrate towards CCL19 was evaluated in an in vitro migration assay. In contrast to vehicle-treated moDC, SFA strongly suppressed moDC migration towards CCL19 (Figure 7 A). Since maturing DCs express the CCL19 ligand CCR7 that directs migration of DC towards lymph nodes, we analysed CCR7 expression after SFA treatment (Figure 7 B, C). Interestingly, SFA administration did not interfere with CCR7 up-regulation indicating that SFA's inhibitory effects on moDC migration were CCR7-independent.

### SFA inhibits the expression of multifunctional molecule CD38

CD38 is an ectoenzyme and signalling receptor and was reported to represent a novel human DC marker [26]–[27]. CD38 is important for innate and adaptive immune responses by regulating DC migration and pro-inflammatory cytokine expression [26]–[27]. Our microarray experiments indicated that SFA inhibited CD38 gene expression (coefficient −2.20949714, p = 1.06*10^−07^). Given the fact that SFA efficiently inhibited moDC migration in a CCR7-independent manner and previous reports demonstrated that SFA can abrogate IL-12 production in human DCs [10]–[11] we questioned whether SFA is able to suppress surface CD38 expression on maturing human moDCs. Flow cytometry analysis with CD38 mAb indicated that SFA caused a significant inhibition of CD38 expression compared to controls and CD38 expression decreased dose dependent after SFA-treatment (Figure 7 D). Interestingly, in contrast to SFA, CsA did not suppress CD38 expression (Figure 7 D).

### Short-term SFA treatment inhibits DC migration in vivo

**Figure 8:**
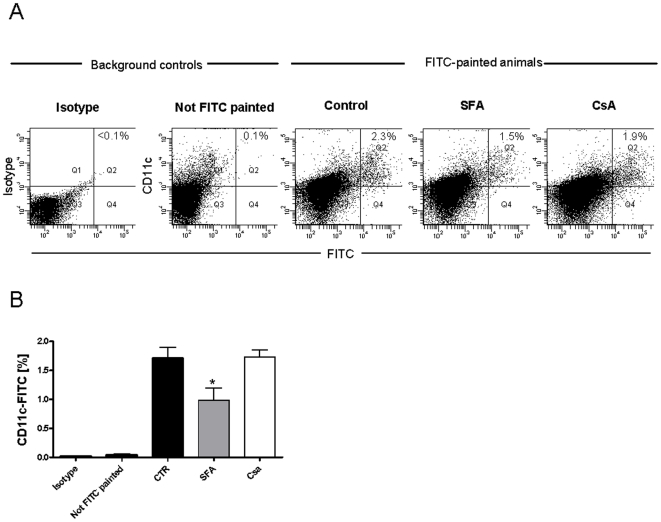
In vivo administration of SFA inhibits migration of FITC-labelled CD11c+ DCs. Mice were injected i.p. with SFA (10 mg/kg/day, 2days), CsA (10 mg/kg/day, 2 days) or vehicle. On day two, abdomen of mice were FITC-painted. The inguinal lymph nodes were removed 24 h later and CD11c+FITC+ migrated cells quantitated by flow cytometry. (A) Dotplot analysis of CD11c+ FITC+ DCs. Numbers indicate percentages of CD11c+FITC+ DCs. (B) Mean (± SEM) numbers of CD11c+ FITC+ DCs in SFA-injected versus vehicle-treated controls. Results are representative for n = 3–5 independent experiments. *p<0.05 versus vehicle.

To investigate the in vivo activity of SFA on DC migration we used the FITC-skin-painting model [28]. We treated animals in vivo with SFA and studied the migration of skin resident CD11c+ DC to the inguinal lymph node. Resident DCs were mobilized by FITC-painting of the shaved abdomen. We created a time course of percentage changes in DC numbers in the inguinal lymph node after FITC-painting. The percentage numbers of DC peaked at 24 h and then decreased. Animals received two i.p. injections of SFA, CsA (10 mg/kg/day) or vehicle 24 hours before and on the day of FITC-skin-painting. Inguinal lymph nodes were prepared and migratory CD11^+^FITC^+^ skin DC quantified after 24 h by flow cytometry (Fig. 8 A, B).

## Discussion

Sanglifehrins represent novel immunosuppressive agents that have been reported to suppress key functions of DCs [9]–[11]. We and others have reported that SFA inhibits bioactive IL-12p70 production, macropinocytosis as well as receptor-mediated endocytosis in human and murine DCs. Transplant experiments indicated that addition of SFA to CsA efficiently suppresses graft arteriosclerosis in comparison to CsA monotherapy suggesting that SFA may represent a novel class of immunophilin-binding agents [11].

However, a disadvantage of previous studies is the fact that they have focused on selected molecules or selected functional aspects thereby restricting the possibility to discover novel mechanisms of action. Accordingly, the aim of the present study was to use a systematic genome-wide approach in order to reveal novel immunobiological effects of SFA on human DC. Secondly, identification of molecules being most specifically suppressed by SFA in comparison to the related molecule CsA may help to elucidate the mechanism of action.

The results presented here indicate that SFA impairs DC-mediated immunity in a so far unrecognized manner that is DC chemokine expression and migration. Importantly, SFA's inhibitory effects can be demonstrated on two different functional levels such as direct chemokine expression inhibition and subsequent impaired attraction of CD4 helper T cells as wells as DC migration inhibition towards recombinant CCL19. Accordingly, we have found that SFA, in contrast to CsA, does not only inhibit mRNA and protein expression of a number of chemokines, including CCL5, CCL17 and CCL19 but additionally suppresses CD38 mRNA and DC surface expression. Thus, SFA's effects on DC are unique in direct comparison to the related cyclophilin-binding immunosuppressant CsA. The latter results provide a rationale for the explanation of reduced migration of SFA-exposed moDCs against recombinant CCL19. CD38 has been reported to be required for the migration of mature DC against recombinant CCL19 [29].

Furthermore, CD38 inhibition by SFA provides additional insight into recent reports demonstrating SFA's capacity to abrogate bioactive IL-12 production in vitro and in vivo. CD38 has been shown to be functionally involved in IL-12 production and IL-12 secretion has been demonstrated to be restored upon CD38 ligation by agonistic anti-CD38 mAbs [26]–[27].

However, it is difficult to assess the specific role of CCL19 inhibition because SFA exerts pleiotropic effects both on chemokine expression and chemokine reponsiveness. Furthermore, CD38 suppression in moDC by SFA may represent only one possible explanation for reduced DC migration but the results do not provide formal evidence for a direct link between CD38 and reduced chemokine expression or responsiveness. Notably, besides migration, CCL19/CCl21 chemokines have been correlated with autoimmunity and immune suppression indicating an important additional role balacing immunity and tolerance [30]–[31].

SFA's effects on CCL5, CCL17, CCL19 and CD38 expression are likely to be independent of cyclophilin-binding since preincubation with a 100-fold molar excess of CsA did not abrogate SFA's inhibitory effects. These findings are in agreement with Zenke et al. [5], who demonstrated that SFA's activity in the MLR is not abrogated in the presence of a 10-fold molar excess of the cyclophilin-binding nonimmunosuppressive derivative, 4-Cs. These findings provided additional insight into SFA's effects to inhibit chronic graft vasculopathy in CsA-treated recipients [11]. Chronic graft vasculopathy is characterized by continuous intimal proliferation and infiltration of leukocytes [32]. The infiltration and activation of leukocytes is mediated by chemokines that are believed to play a critical role in the immunopathology of this process [33]–[34]. Suppression of DC chemokine expression and DC migration by SFA is likely to promote SFA's capacity to inhibit graft vasculopathy.

In conclusion, this first systematic genome-wide study revealed a novel anti-inflammatory mode of action of SFA being different from the related agent CsA. The suppressive activity of SFA with regard to DC chemokine expression and migration in addition to its inhibitory effects on DC antigen uptake and DC bioactive IL-12 production identifies this immunophilin-binding agent as a novel partner for combination with potent T-cell inhibitors. Furthermore, with respect to the development of novel cell migration inhibitors targeting either chemokine receptors, selectin receptors or integrin receptors [35], SFA seems to represent an attractive combination partner to potentiate the anti-inflammatory activity of these novel agents. Since this study was focused on the systematic analysis of SFA's effects on human moDCs, further studies are necessary to analyse the effects of SFA on chemokine expression in T and B lymphocytes.

## Materials and Methods

### Ethics statement

The in vitro studies of human blood samples were approved by the ethic study board of the University Hospital Gießen (File Nr 05/00) and the animal experiments were approved by the animal ethics review board of the Regierungspräsidium Gießen (File GI 20/8 Nr 49/2006).

### Compounds

SFA and N-Methyl-Val-4-cyclosporin (4-Cs) were provided by Novartis Pharmaceuticals (Basel, Switzerland). Stock solutions were prepared in absolute ethanol (vehicle) and the control DCs were treated with drug vehicle. Dexamethasone and Cyclosporin A were provided by Sigma-Aldrich (Seelze, Germany) and dissolved in vehicle. The drugs were used at the indicated concentrations and time points. The stock solutions were diluted on the day of experiment with culture medium.

### Generation of moDC

Human PBMC were isolated from buffy coats of healthy blood donors by Ficoll-Paque (Amersham Bioscience, Uppsala, Sweden) density gradient centrifugation. CD14^+^ monocytes were purified (>95%) using CD14 immunomagnetic microbeads (Miltenyi Biotec, Bergisch-Gladbach, Germany) and 1×10^6^ cells per ml were cultured in six-well flat-bottom plates, in 3 mL of DC medium, comprising RPMI 1640, L-glutamine, penicillin/streptomycin, sodium-pyruvate, hepes, nonessential amino acids, 10% heat-inactivated FCS Gold (PAA Laboratories, Linz, Austria), 1000 IU/ml recombinant human (rh) GM-CSF (Promo Cell, Heidelberg, Germany), and 1000 IU/ml rhIL-4 (Strathmann Biotech GmbH, Hamburg, Germany). On day 6, CD1a^+^ DC represented >90% of cultured cells. The in vitro studies of human blood samples and the animal experiments were approved by the Institutional Review Board.

### CD4^+^T cell isolation

CD4^+^-T cells were positively selected with magnetic beads and subsequently sorted with autoMACS (Miltenyi Biotec, Bergisch Gladbach, Germany). Reagents were used according to the manufacturer's instructions. Purity of positively selected T cells was >98% determined by flow cytometry. 1×10^6^ cells per ml were cultured in six-well flat-bottom plates, in 3 mL of medium, comprising RPMI 1640, L-glutamine, penicillin/streptomycin, 10% heat-inactivated FCS Gold (PAA Laboratories, Linz, Austria).

### DC stimulation, RNA Isolation and cDNA Microarray Analysis

On day 5, 1 µM SFA or vehicle (absolute ethanol) was added to the DC medium. After one hour 1 µg/mL Lipopolysaccharid (LPS, *Escherichia coli* 026:B6; Sigma-Aldrich, Taufkirchen, Germany) was added to the culture. After 12 hours stimulation, the RNA of 1.5×10^7^ cells was isolated by using the Qiagen RNeasy Mini Kit (Hilden, Germany) according to the manufacturer's instructions.

The RNA from four donors was pooled and the RNA was stored at −70°C until use. A total of n = 28 donors were analyzed by microarray. The RNA quality was confirmed with RNA 6000 Nano LabChips (Agilent Technologies, Palo Alto, USA). 1 µg RNA was amplified with Low RNA Input Fluorescent Linear Amplification Kit (Agilent Technologies, Palo Alto, USA) and labeled with Cyanin 3- or Cyanin 5-CTP (Perkin Elmer, Rodgau, Germany). cRNA of SFA and vehicle cRNA was mixed and 40 µg cRNA was used for hybridization with whole human genome oligo Microarray G4112A (Agilent Technologies, Palo Alto, USA). A total number of 7 arrays were hybridized, each with a pool of RNA from control samples and a pool of RNA from stimulated samples (dual color with balanced between-sample dye-swap). Hybridization and washing was done following the Agilent protocol. Images were scanned with the Axon 4100A (Molecular Devices, Sunnyvale, USA) and processed with GenePix 5.0. Data analysis was done with R software version 2.10.1 (http://www.R-project.org) using Limma [36]. Intensity values were corrected for local background before calculation and loess normalization of the M/A values. Genes were ranked for differential expression by moderated t-statistics. P values were corrected for multiple testing using the method of Bonferroni and Holm.

### DC stimulation and detection of cytokine and chemokine production by ELISA

For the chemokine expression analysis SFA, CsA and Dexamethasone were added at day 5 for 4 hours at the indicated concentrations. Subsequently, human moDCs were incubated at 2×10^6^/ml in 96-well plates in DC medium with cytokines and stimulated for 12 h with 100 ng/ml LPS (*Escherichia coli* 026:B6; Sigma-Aldrich, Taufkirchen, Germany). Phenotypic maturation of moDC after LPS stimulation was controlled by flow cytometry analysis of surface CD83, CD86 and MHC-II (HLA-DR) expression (Table 2). Human CXCL10 and CXCL9 were measured using BD OptEIA ELISA sets (BD PharMingen, San Diego, USA). The Chemokines CCL1, CCL5, CCL17 and CCL19 were measured using R&D Systems Developmental DuoSets (R&D Systems, Wiesbaden-Nordenstadt, Germany) according to the manufacturer's instructions.

**Table 2:** Phenotypic maturation of LPS-matured moDC.

Surface expression*	moDC (± SEM)	LPS-matured moDC (± SEM)
CD83	2351 (±77)	3971 (±443)
CD86	993 (±357)	8158 (±716)
MHC-class II (HLA-DR)	6784 (±872)	14910 (±868)

*Median fluorescence intensity determined by calibrated flow cytometer before and after LPS stimulation (100 ng/ml LPS; 12 h; n = 4).

### CCR7 and CD38 analysis by flow cytometry

Human moDCs were treated with SFA or vehicle at day five. After four hours, LPS was added to the culture for additional 24 h. The drugs were used at the indicated concentrations. Surface staining was performed with CD1a-FITC, CD14-PE, CD38-APC or CCR7-APC (BD Biosciences, R&D Systems) and appropriate isotype controls (BD Biosciences, R&D Systems) according to the manufacturer's instructions.

### In vitro migration assay

Migration was analysed by using a 24-well microchemotaxis chamber technique with polycarbonate transwells (5 µm pore size; Corning Costar, NY, USA) [37]. 1×10^6^ moDCs or CD4+ T cells were suspended in supplemented medium. 100 µl were placed in the upper well. moDC migrated towards 100 ng/ml CCL19 or in other experiments human moDCs and T cells migrated towards cell-free supernatant of SFA-exposed moDC or vehicle-treated moDCs in the lower well. For the “SFA carry over control” supernatant of vehicle-treated moDC was used and spiked with 1 µM SFA. We treated human moDCs with 1 µM SFA or vehicle for 4 hours and collected the supernatant after 12 h stimulation with 100 ng/ml LPS. The transwells were incubated for 4 hours at 37°C in a 5% CO_2_ moist atmosphere. Migrated cells were quantitated with Leukocount-Kit (BD Biosciences, San Diego, USA) on a FACSCalibur flow cytometer (BD Biosciences, San Jose, USA).

### In vivo migration of DCs after Sanglifehrin A treatment

8–10 wk-old female C57BL/6NCrl mice were treated with SFA or CsA (10 mg/kg/for 2 days). Drug stock solution was diluted freshly in 2.5% Polysorbate 80, 51% PEG300 (Sigma-Aldrich) and 46.5% sterile water. On day two, the abdomen was shaved and painted with 200 µl FITC (10 mg/ml), in a 50∶50 (vol/vol) acetone-dibutylphtahalate mixture, as described by Macatonia et al. [28]. Inguinal lymph nodes were obtained at indicated time points, mechanically disaggregated and passed through a cell mesh. Cell suspensions were stained with CD11c-APC mAb and quantitated by FACSCalibur (BD Biosciences, San Jose, USA).

### Statistical analysis

Statistical analyses were performed using the Mann Whitney U test. All tests were performed two-tailed. A probability of <0.05 was considered significant. Analyses were performed using the SPSS software version 16.0 (SPSS Inc, Chicago, Ill.). Microarray results were analyzed as described above.
